# Recent Advances in Integrative Multi-Omics Research in Breast and Ovarian Cancer

**DOI:** 10.3390/jpm11020149

**Published:** 2021-02-19

**Authors:** Christen A. Khella, Gaurav A. Mehta, Rushabh N. Mehta, Michael L. Gatza

**Affiliations:** Rutgers Cancer Institute of New Jersey, New Brunswick, NJ 08903, USA; cak241@scarletmail.rutgers.edu (C.A.K.); gam160@cinj.rutgers.edu (G.A.M.); rnm53@scarletmail.rutgers.edu (R.N.M.)

**Keywords:** genomics, proteomics, breast, ovarian, cancer

## Abstract

The underlying molecular heterogeneity of cancer is responsible for the dynamic clinical landscape of this disease. The combination of genomic and proteomic alterations, including both inherited and acquired mutations, promotes tumor diversity and accounts for variable disease progression, therapeutic response, and clinical outcome. Recent advances in high-throughput proteogenomic profiling of tumor samples have resulted in the identification of novel oncogenic drivers, tumor suppressors, and signaling networks; biomarkers for the prediction of drug sensitivity and disease progression; and have contributed to the development of novel and more effective treatment strategies. In this review, we will focus on the impact of historical and recent advances in single platform and integrative proteogenomic studies in breast and ovarian cancer, which constitute two of the most lethal forms of cancer for women, and discuss the molecular similarities of these diseases, the impact of these findings on our understanding of tumor biology as well as the clinical applicability of these discoveries.

## 1. Introduction

Each year more than 1.8 million people are diagnosed with cancer in the United States including more than 270,000 breast cancer patients and 21,000 ovarian cancer patients [1]. Despite advances in diagnostic tools, predictive biomarkers, and new therapies over the past 20 years which have led to declining mortality rates, more than 285,000 people will die each year in the US due to their disease, including more than 50,000 breast and 13,000 ovarian cancer patients [1]. Enormous clinical variability, including disease progression and response to therapy has been shown to exist for most forms of cancer. These observed differences are driven, in part, by underlying genetic, genomic, and proteomic alterations unique to each patient [2,3,4,5]. In essence, cancer is not a single disease but rather a collection of genetically driven malignancies affecting a given tissue. As a result, all tumors, even within a given tissue type, cannot be treated equally [6,7,8,9,10,11,12]. New tools, therapies, biomarkers, and treatment strategies are being developed or will need to be developed, to identify and target those mutations and/or signaling pathways essential for each tumor to improve clinical outcome and quality of life for each patient.

The underlying genetic heterogeneity within human cancers creates several challenges both clinically and from a basic science perspective. From a mechanistic standpoint, variability in patterns of genomic and proteomic alterations create a challenge in separating the key drivers of oncogenic signaling, tumor development, and progression from those mutations that are tumor-promoting but non-transforming or that do not directly contribute to tumorigenesis (i.e., passenger mutations). This is essential as not all mutated or aberrantly expressed genes are required for tumorigenesis nor do they equally contribute to therapeutic response [13,14]. As a result, there is a need to develop tools and approaches to understand the interplay between altered genes and to determine how these genes or proteins promote aberrant signaling, including the identification of novel signaling networks and cellular processes that contribute to tumor growth and progression. Finally, utilizing the compendium of alterations across a given tumor type, we must develop approaches to identify novel therapeutic targets and determine predictive biomarkers to recognize patients that are likely to benefit from specific therapeutic regimens.

Over the past 20 years, beginning with the sequencing of the human genome to the more recent development of next-generation sequencing (NGS), advances in genomics, proteomics, and systems biology have allowed us to begin to catalogue, visualize, compare and dissect patterns of DNA mutations and copy number alterations, mRNA and miRNA expression patterns, protein and phosphorylated protein expression and epigenetic alterations between individual patients, across specific forms of cancer and between malignancies affecting different tissues [2,3,4,5,15,16]. These studies, coupled with functional genomic studies, have begun to identify and provide insight into key drivers of oncogenic signaling, mediators of specific tumor characteristics, including response to therapy, and identify novel treatment strategies. In this review, we will examine historical and recent advances in genome and proteome-wide analyses in breast and ovarian cancer and discuss the impact of these findings on our understanding of tumor biology as well as the clinical applicability of these discoveries. 

## 2. Clinical Characterization of Breast and Ovarian Cancer

Breast cancer is the most commonly diagnosed and the second leading cause of cancer-related mortality for women in the United States [1]. While it is estimated that approximately 50,000 women in the US and 522,000 women worldwide will die from this disease annually, survival rates have steadily increased by over 40% over the past 30 years [1,17]. Currently, more than 98% of patients diagnosed with early stage disease are expected to live for at least 10 years and the current 5-year survival rate is ~90% across all stages [1,17,18,19]. These improvements can be attributed, in part, to increased early detection from earlier screening and improved imaging technology as well as the development of novel therapeutic regimens incorporating chemotherapeutics, targeted therapies, radiation, surgery, and immunotherapy [20,21,22]. Despite these advances, the prognosis for patients with locally advanced and metastatic disease remains poor. Patients with advanced metastatic disease have a 5-year survival rate of less than 30% and a significant percentage of patients whose tumors are inoperable and/or refractory to current therapies will succumb to their disease within 5 years irrespective of tumor stage at diagnosis [18].

Part of the challenge in developing effective treatments for this disease lies in the molecular and clinical heterogeneity that exists between each patient’s tumor. Clinically, breast tumors are classified based on morphological features with ~70% of tumors being classified as invasive ductal carcinomas (IDC), ~15% categorized as invasive lobular carcinoma (ILC), and the remaining tumors regarded as rare subtypes [18,23]. Prognosis and treatment strategies are largely dictated by classical histopathologic features including tumor size, histological grade and stage, lymph node status, and the expression of hormone receptors or HER2 (human epidermal growth factor receptor 2) status [18,19]. Among the histological subtypes, estrogen receptor (ER), progesterone receptor (PR), and HER2 status can be used to further delineate patients into ER+/PR+ (60–70% of patients), HER2+ (10–20%), and triple negative breast cancer (TNBC, 15–20%). However, differences in the prevalence of these histological subtypes are seen between women with different ancestries. Notably, women of African American decent have a higher incidence of TNBC when compared to American women of European ancestry (36.3% vs. 13.7%) [1,24,25]. Importantly, these biomarkers are used to direct current standard-of-care treatments with endocrine-based therapies comprising the core of therapeutic regimens to treat hormone receptor-positive (HR+) breast tumors; HER2-family inhibitors forming the foundation for therapies used to treat HER2+ patients [26], and multi-agent cytotoxic chemotherapies providing the basis for the treatment of TNBC patients [27,28]. While endocrine-based therapies result in remission in the majority of patients with HR+ tumors, approximately 30–50% of patients manifest primary or acquired resistance [29,30]. Recent studies have reported that the emergence of hormone therapy resistance in ER+ breast cancers can arise through four predominant mechanisms including ESR1 mutations (18%), altered MAPK signaling (13%), MYC or transcription factor activation (9%), and other/unknown factors (60%) [31]. The findings of multiple clinical trials have resulted in FDA and international approval for use of the mTOR inhibitor everolimus in conjunction with exemestane for the treatment of patients with advanced or metastatic ER+, PR+, HER2-negative, PIK3CA mutant tumors [32]. Likewise, the PI3K inhibitor alpelisib has been approved for the same patient population in combination with fulvestrant [33]. More recently, CDK4/6 inhibitors palbociclib, ribociclib, and abemaciclib have been approved and, in conjunction with hormone therapy, have become the primary treatment regimen for HR+/HER2- treatment naïve or hormone therapy treated metastatic breast cancer patients [34]. Finally, the treatment of TNBC tumors has begun to evolve to include immune checkpoint inhibitors while patients with BRCA mutations are treated with PARP inhibitors in conjunction with chemotherapy [28,35,36]. 

In contrast to breast cancer, ovarian cancer is a less frequently diagnosed malignancy with approximately 21,750 women in the US and 250,000 women worldwide being diagnosed with this disease annually [1]. Unfortunately, however ovarian cancer is the most lethal form of gynecological cancer with an estimated 13,940 women dying from this disease in the United States and more than 150,000 women dying worldwide in 2020 [1]. This translates to a death-to-case ratio of approximately 64%, far outpacing the lethality of breast cancer [1]. 

Similar to breast cancer, the clinical complexity of ovarian cancer is due, in part, to histological and molecular heterogeneity. Ovarian tumors are classified into four major classes: high (70%) and low (4.1%) grade serous, endometrioid (8.3%), clear cell (9.5%) and mucinous (3.2%) carcinoma [37,38,39]. Of note, a study by Beckmeyer-Borowko and colleagues showed that non-Hispanic Black ovarian cancer patients were more likely to be diagnosed with stage four HGSOC, clear cell or mucinous carcinomas when compared to non-Hispanic White patients [40]. Beyond these classifications, ovarian epithelial tumors have been divided into Type I and Type II tumors [41,42,43,44]. Type I tumors typically encompass low-grade and indolent tumors including low-grade serous, low-grade endometrioid, clear cell, and mucinous carcinomas that tend to present as stage I tumors while Type II tumors include more aggressive and high-grade tumors including high-grade serous, high-grade endometrioid, malignant mixed mesodermal tumors, and undifferentiated carcinomas [42]. Genetically, Type I and II tumors are characterized by specific mutations: mutations common to Type I tumors include KRAS, BRAF, ERBB2, CTNNB1, PTEN, PIK3CA, ARID1A, and PPP2R1A, while Type II tumors have a high frequency of TP53 mutations (>95%) as well as mutation or aberrant expression of BRCA1 or BRCA2 [3,42,43,44]. Importantly, these mutations appear to be largely confined to each subtype with Type II tumors rarely expressing Type I mutations and Type I tumors being largely wild-type for TP53, except for low-grade mucinous tumors (~25%) [45].

While more than 92% of Stage I ovarian cancer patients are successfully treated, only 15% of patients are diagnosed with the early stage disease [1]. High-grade serous ovarian cancer (HGSOC) is the most prominent form of ovarian cancer and accounts for 70% of ovarian cancer-related deaths [46,47]. Although most patients will initially respond favorably to standard-of-care cytoreduction surgery followed by platinum- and taxane-based treatment, approximately 80% will eventually relapse and develop resistance in late stage disease [39,48,49,50,51]. In addition, 25% of patients are inherently resistant to standard-of-care therapy and demonstrate disease progression within six months of treatment [52]. More recent studies have determined that HGSOC tumors are characterized by homologous recombination deficiencies (HRD) which render these tumors sensitive to PARP inhibition [53,54,55,56]. As such, PARP inhibitors (olaparib, rucaparib, niraparib) were FDA approved for treatment of platinum-sensitive recurrent, BRCA mutated, and HRD-positive epithelial ovarian cancer [34,56,57,58,59,60,61]. In addition, olaparib in combination with bevacizumab has been approved for the treatment of patients with advanced epithelial ovarian cancer. This combination treatment nearly doubled the progression-free survival in HRD-positive tumors when compared to bevacizumab alone [62]. Finally, clinical trials examining the impact of multiple novel combinatorial strategies, including VEGF inhibitors (VEGFi) in combination with PARP inhibitors (PARPi) as well as anti-PD-1 inhibitors, alone or in combination with VEGFi and/or PARPi, are ongoing [63]. While significant advances in the molecular characterization of ovarian cancer have led to a better understanding of this disease, the prognosis has not significantly improved over the past several decades; poor prognosis is attributed to lack of early detection and resistance (inherent and acquired) to platinum-/taxane-based therapies [3,46]. 

Despite the inherent differences in clinical manifestation between breast and ovarian cancer, a portion of these malignancies are intrinsically linked, as women with specific inherited germline mutations including BRCA1, BRCA2, PALB2, TP53, CDH1, and PTEN have an increased lifetime risk of developing either disease [64,65]. BRCA1 or BRCA2 mutations are the most prevalent cause of high penetrance inherited breast or ovarian cancers and have been shown to affect patients irrespective of race or ethnicity. Overall, the rate for germline BRCA1 or BRCA2 mutations is relatively low with 4–6% of breast and 8–15% of ovarian tumors expressing one of these mutations [66,67,68,69,70,71,72,73]. However, it is estimated that 39–63% of women with a BRCA1 mutation will develop ovarian cancer while 46–87% will develop breast cancer by age 70. Likewise, BRCA2 mutation carriers are strongly predisposed to develop ovarian (17–27%) or breast (38–84%) cancer [65,74,75,76,77]. Clinically, BRCA1/2-mutated breast tumors tend to be classified as TNBC invasive ductal carcinoma with high nuclear grade while BRCA1/2-mutated ovarian tumors are predominantly classified as HGSOC [78,79,80]. Although BRCA-mutated breast and ovarian tumors are often highly aggressive, a number of studies suggest that these patients may achieve a slightly better short-term therapeutic response (2–3 year overall survival) compared to patients with wild-type BRCA1 or BRACA2, as these tumors may be more responsive to DNA-damaging drugs; however, long-term survival and/or progression-free survival differences remain unclear [65,79,80,81,82]. 

While inherited breast and ovarian cancers have similar features, including response to specific inhibitors, as we will discuss below, non-familial ovarian and some subsets of breast tumors also demonstrate striking genome- and proteome-wide similarities including somatic mutations, patterns of copy number alterations, and expression of specific genes, proteins and signaling pathways. By utilizing this information, more recent treatment strategies for breast and ovarian cancers have begun to incorporate targeted therapies in conjunction with standard-of-care treatments [18,19,21,83]. While these novel regimens have improved clinical response and quality of life, as we have discussed, these treatments are often limited to patients with specific genomic alterations or clinical subtypes and not all patients will respond equally. These observations highlight the need to not only develop new, more effective therapies but also illustrate that it is necessary to develop a genome- or proteome-wide portrait of the underlying molecular heterogeneity of each of these diseases. Gaining a more complex view of the underlying biological mechanisms driving disease development, progression, and response to treatment will allow investigators to identify and develop biomarkers that will enable the design and evolution of treatment regimens based on the underlying biology of a given patient’s tumor. 

## 3. Molecular Classification and Characterization of Breast Cancer

**Figure 1:**
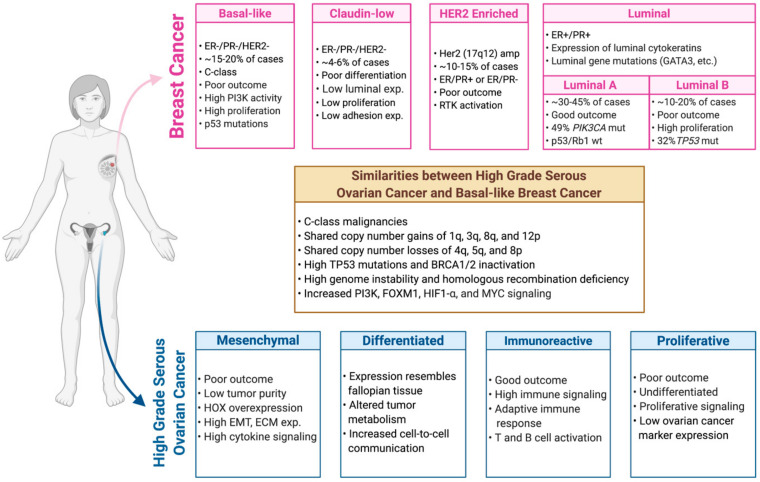
Gene expression-based classification of breast and ovarian cancers. The major molecular classifications of breast and ovarian cancers are depicted here. Further highlighted are the molecular similarities between high grade serous ovarian and basal-like breast cancer.

Seminal studies by Perou and colleagues used microarray-based gene expression profiling and unsupervised hierarchical clustering to identify a 496 intrinsic gene list that defined five molecularly distinct subtypes of breast cancer [84,85]. These subtypes clustered largely along the estrogen receptor status with ER-positive tumors being classified into luminal A (LumA) or luminal B (LumB) subtypes while ER-negative tumors were classified as HER2 enriched (HER2E), basal like, or normal like [84,85,86,87] (Figure 1).

The ER-positive luminal tumors express luminal cytokeratins 8 and 18 and are enriched for genes expressed by breast luminal epithelial cells, including GATA3, FOXA1, ESR1, and MYB. Among luminal tumors, LumB tumors are defined by higher expression of proliferation-related genes, high genomic risk, and poorer clinical outcome than LumA tumors. HER2E tumors are predominantly ER negative, characterized by the amplification of the HER2 gene on chromosome 17q12, and are associated with poor prognosis and increased risk of metastasis. The basal-like subtype is largely synonymous with triple negative breast cancer (TNBC). These tumors express basal epithelial cell markers keratin 5/6 and are characterized by enrichment of the genes expressed by breast basal or myoepithelial cells [2]. Basal-like tumors represent the most diverse subtype of breast cancer and are associated with high proliferation rates, high mutational burden, higher risk of metastasis, and poor survival rates [85,86]. Finally, the normal-like breast cancer subtype has also been described and is typified by high expression of genes known to be expressed by basal epithelial cells and adipose cells. However, the biological relevance and clinical importance of this subtype remains unclear [84,85,86,87]. 

The association between molecular subtypes and disease-specific outcomes demonstrate that tumor cell response to treatment is not determined by anatomical prognostic factors but rather inherent molecular features, indicating the potential clinical value of these expression-based patient classifications [84,85]. However, the ‘intrinsic’ gene set used by Perou and group to experimentally categorize patients was not readily employable in the clinic due to its relatively large size [84,85,86,87]. Utilizing microarray data and several minimization methods, Parker et al. developed a reliable 50-gene signature to identify breast cancer intrinsic subtypes [88]. Combined with common histologic criteria, such as tumor grade and pathologic staging, the 50-gene signature (PAM50) provided significant prognostic and predictive value through classification and generating risk-of-relapse (ROR) scores for all patients [88]. While the clinical implications of the PAM50 subtype predictor remain to be fully resolved, the Prosigna assay, which is derived from the initial intrinsic analyses, is used clinically to help predict risk of relapse and to guide therapeutic intervention [89,90,91].

The recent advances in gene expression profiling platforms have led to the identification of additional molecular subtypes, further defining the biological and clinical heterogeneity of breast cancer. The claudin-low subtype was identified to be predominantly triple negative and poorly differentiated subgroup of breast tumors which are enriched for cancer stem cell-like genomic signatures and immune response genes [92,93]. These tumors are characterized by low expression of luminal genes, proliferation genes, and genes involved in tight junctions and cell–cell adhesion [92,93]. More recent gene expression studies employed by Lehmann et al. initially categorized TNBC tumors into six molecular subtypes, including BL1 and BL2 (basal-like), immunomodulatory (IM), mesenchymal (M), mesenchymal stem-like (MSL), and luminal androgen receptor (LAR) [94]. This classification has since been further refined to include the four (TNBCtype-4) tumor-specific subtypes (BL1, BL2, M, and LAR) and exclude the IM and MSL subtypes due to the identification of transcripts from infiltrating lymphocytes and tumor-associated stromal cells, respectively [95]. The TNBC type-4 subtypes demonstrated significant differences in histopathology, grade, and local and distant disease progression [95]. These subtypes were characterized by unique identities of pathway activation which stimulated the use of known inhibitors and therapies to exploit signaling vulnerabilities, exhibiting early evidence of clinical applicability [94,95].

Decomposing vast amount of information from profiling studies represents a key step in developing patient-specific therapeutic regimens. In light of this, pathway signatures were developed as an underlying platform to provide a functional interpretation of the gene expression data within each subtype and further dissect the heterogeneity of breast cancer [96]. Integrated analysis using gene expression and pathway activation probabilities contributed to stratifying tumor subtypes and characterizing distinct clinical and biological features [96,97,98,99,100]. Along these lines, Gatza et al. utilized pathway activation probabilities that reflect in vivo activity levels to identify subgroups that reflect the status of important signaling pathways in breast tumors [97]. These subgroups corresponded to the intrinsic subtypes and exhibited distinct patterns of pathway activation, DNA copy number changes as well as clinical and biological characteristics [97,98].

While microarray-based gene expression profiling of breast tumors has been able to distinguish tumor subgroups and begin to define underlying biological and clinical diversity, these studies were limited in their ability to create true “molecular portraits” of breast cancer. Large-scale integration of multiple proteogenomic platforms through The Cancer Genome Atlas (TCGA) project provided a more comprehensive view of breast cancer heterogeneity and underlying biology. The TCGA project (*n* = 1072) used data from six different high-throughput technology platforms, including mRNA expression microarrays (and mRNA sequencing), DNA methylation, genomic DNA copy number arrays, microRNA sequencing, whole-exome sequencing, and reverse-phase protein array (RPPA) to examine specific genetic, epigenetic, and proteomic alterations in breast cancer and to link these alterations to clinical data and characteristics [2]. Intriguingly, while the overall patterns of proteogenomic alterations were found to be variable amongst patients, including between subtypes, intra-subtype variation was limited. Remarkably consistent patterns of genomic and proteomic alterations were found to be associated with each of the mRNA-based PAM50 subtypes.

Luminal tumors are characterized by an increased frequency and diversity of significantly mutated genes in addition to a lower frequency of copy number alterations [2,101,102]. These tumors exhibit increased mutations in luminal genes including GATA3 and FOXA1, as well as genes belonging to the p38-JNK pathway (MAP3K1 and MAP2K4), which were mutated in a mutually exclusive manner. PIK3CA, which is the most frequently mutated gene in breast cancer, was predominantly altered in luminal tumors and was mutated at a much higher frequency in LumA (45%) relative to LumB (29%) tumors. Despite the high frequency of activating PIK3CA mutations in LumA subtype tumors, the PI3K/AKT signaling axis has not been shown to be consistently upregulated in these tumors. In contrast to LumA tumors, LumB tumors are characterized by higher inactivation of the TP53 pathway associated with a higher rate of mutation in the TP53 gene, loss of ATM2, and MDM2 amplification [2]. More recent integrative analysis using 52 gene expression signatures that measure oncogenic signaling pathways identified a limited number of genes that are amplified and overexpressed in aggressive luminal subtype tumors. Among these genes, a subset (FGD5, METTL6, CPT1A, DTX3, MRPS23, EIF2S2, EIF6, and SLC2A10) was found to be essential for cell growth and, in some instances, correlated with clinical outcome [99,103,104,105]. This study further suggests that not only do LumA and LumB tumors express unique mutation profiles, but that these alterations result in distinct patterns of oncogenic signaling beyond differences in proliferation.

The HER2E subtype is characterized by high amplification of the HER2 amplicon (80%) on chromosome 17q12. These tumors can be either ER negative or positive and demonstrate increased expression of the HER2 oncogene as well as other genes on the 17q12-amplicon, including GRB7. However, not all clinically defined HER2-positive tumors are categorized into this subtype, as some ER+/HER2+ tumors demonstrate increased expression of specific luminal genes (i.e., GATA3, BCL2, and ESR1) and cluster largely into the LumB subtype. TP53 (72%) and PIK3CA (39%) mutations are highly enriched in this subtype and show significantly higher expression and activation of receptor tyrosine kinases such as FGFR4, EGFR, and HER2 [2].

Basal-like breast cancers represent the most heterogeneous subtype with a high frequency of TP53 mutations which are present in an overwhelming 80–90% of tumors. In addition to TP53 truncating mutations, these tumors are characterized by loss of RB1 and BRCA1 along with amplification and hyperactivation of the MYC and FOXM1 genes. Increased activation of PI3K/AKT signaling, relative to other subtypes is a distinguishing feature of basal-like tumors despite a low incidence of PIK3CA (9%) mutations. Expression of keratins 5, 6, and 17 and cell proliferation genes are significantly upregulated in these tumors owing to the increased expression of FOXM1 as a transcriptional driver of this gene signature [2]. 

Similar multiplatform analysis was also conducted to provide molecular context to invasive lobular breast cancer, which is the second most commonly diagnosed invasive breast cancer and comprise approximately 10–15% of all cases. Despite histological differences, invasive lobular carcinomas (ILC) and ER+ invasive ductal carcinomas (IDC) patients have historically been treated similarly, emphasizing the need to more robustly understand the molecular underpinnings of the disease for better therapeutic interventions [106]. Multiplatform studies carried out by the TCGA project and Desmedt et al. identified mutations in the E-cadherin (CDH1) gene (63% in ILC vs. 2% in IDC) which is the hallmark feature of ILCs. In addition to CDH1 loss, mutations in PTEN, TBX3, FOXA1, and ESR1 were enriched in ILC relative to IDC tumors. Mutations in PIK3CA were reported in 48% of ILC relative to 33% of IDC tumors which, along with loss of PTEN function, defines the significant upregulation of PI3K signaling in ILC tumors [23,106]. Transcriptomic analysis identified molecular ILC subtypes which were characterized by unique molecular profiles and clinical outcomes with more proliferative tumors demonstrating a worse clinical prognosis [23,106]. Overall, these multiplatform analyses not only better distinguished between lobular and ductal carcinomas but also identified clinically relevant heterogeneity that may help to better differentiate and treat these carcinomas. In addition to TCGA, the METABRIC (Molecular taxonomy of breast cancer international consortium) study used an integrated clustering approach to examine the genomic and transcriptomic architecture of 2000 breast tumors (along with clinical data) and classify them into 10 integrative clusters (IntClust 1–10) which demonstrate distinct alterations and clinical outcomes [9,107]. Importantly, this classification strategy demonstrated that incorporation of both mRNA and cDNA copy number data identified additional granularity within the PAM50 subtypes as well as molecularly distinct entities based on the underlying genetic alterations. These data, coupled with multi-platform orthogonal analyses performed by TCGA have provided enormous insight into the underlying genetic framework of breast cancer; however, these studies were limited in their ability to associate the genomic and transcriptomic features with the proteome and phosphoproteome that drives the phenotypic characteristics of a tumor. The RPPA platform used by TCGA for quantifying protein abundance and post-translational modifications is limited by antibody quality and an inability to detect mutant protein forms. 

Consistent with this premise, analysis of the proteome and phosphoproteome was performed by the Clinical Proteomic Tumor Analysis Consortium (CPTAC) using mass spectrometry-based analyses to integrate and contextualize genome-scale alterations of 105 tumors and adjacent normal samples [5]. In breast cancer, these analyses resulted in the identification of an average of more than 11,000 proteins and 26,000 phosphosites per tumor significantly extending the previous work from TCGA where only 141 proteins and 31 phosphosites were captured [2,5,23]. Phosphoproteomic analysis informed the translational outcomes of PIK3CA mutations in breast cancer, which often are not correlated with the transcriptional signature of breast tumors. These analyses resulted in the identification of 62 different phosphosites in PIK3CA mutated breast tumors, including *RPS6KA5* and *EIF2AK4*, explaining the activation of the pathway and revealing possible druggable kinases in this pathway [5]. The CPTAC project highlights the need for integrating data across proteogenomic platforms to connect somatic mutations with the activation of various oncogenic signaling pathways in tumors for better therapeutic outcomes. In addition, CyTOF (Cytometry by Time of Flight), has been used for real-time high-dimensional analysis of breast cancer [108,109,110]. For example, a recent study by Ali et al. emphasized the significance of multiplatform analyses when coupled with multidimensional imaging mass cytometry in highlighting the tumor heterogeneity both on tumor-specific and tumor microenvironment levels which in turn affect the tumor evolution, ecosystem and clinical outcomes [109]. Similarly, imaging mass cytometry has been used to generate high-dimensional images of 281 human breast tumor samples in order to identify the spatial architecture, and to define heterogeneity between intra and inter-tumoral cell subpopulations [110].

## 4. Molecular Classification and Characterization of Ovarian Cancer

Similar to studies in breast cancer, studies by Tothill (*n* = 285) [111], the TCGA project (*n* = 489) [3], Helland (*n* = 939) [112], and Konecny (*n* = 174) [113] utilized K-means clustering and non-negative matrix factorization consensus clustering to classify HGSOC into four distinct gene expression-based subtypes. These four molecularly distinct subtypes (Figure 1) were termed immunoreactive, proliferative, mesenchymal, and differentiated based on molecular and clinical characteristics [3]. However, in contrast to molecular subtypes of breast cancer which have clear biological and clinical implications, these relationships do not appear to be as robust in HGSOC.

Mesenchymal tumors have been reported to have the worst clinical prognosis of the four HGSOC molecular subtypes [111,113,114]. These tumors are defined by low tumor purity and demonstrate increased desmoplasia and reactive stromal components, including CD3+ infiltrates [4,111,115]. Phenotypically, these tumors exhibit increased epithelial to mesenchymal transition (EMT), angiogenesis, extracellular matrix (ECM) remodeling, and proteolysis [113,115]. Consistent with these findings, mesenchymal subtype tumors demonstrate increased expression of HOX genes which contribute to development regulation as well as aberrant TGFβ, stromal-associated, wound response, and fos-jun signaling as demonstrated by gene expression signatures [116]. Global proteomic analyses by the CPTAC project further demonstrated that these tumors exhibit increased expression of ECM and cytokine signaling at the protein level [4].

Similar to mesenchymal subtype tumors, immunoreactive tumors are defined by low tumor purity [4]. However, immunoreactive subtype tumors are associated with a good clinical prognosis [111,113,114,115]. While these tumors do demonstrate infiltration of stromal cells, immunoreactive tumors appear to be defined by increased immune signaling, likely due to increased immune cell infiltration [117]. Gene and protein expression profiling studies have reported activation of the adaptive immune response as well as increased T and B cell activation markers, antigen presentation, and chemokine signaling [3,111,113,115]. Consistent with these findings, it was reported that mesenchymal and immunoreactive tumors are more closely related to each other, as compared to the proliferative or differentiated subtypes, despite differences in patterns of signaling network activity and clinical outcomes [113,118]. These similarities are likely due to the low tumor cell purity that is apparent in mesenchymal and immunoreactive tumors, while the distinction between these groups is driven by both underlying tumor biology as well as the composition of infiltrating cell populations in the tumor microenvironment. Consistent with these ideas, recent single-cell RNAseq studies have demonstrated that unique aspects of the tumor microenvironment may define signaling within these subtypes; immunoreactive tumors were shown to have immune-related cell clusters while mesenchymal tumors contained cell clusters enriched for cancer-associated fibroblast signaling [117].

Tumors classified in the proliferative subtype are associated with poor overall survival [106,107,109]. In contrast to immunoreactive or mesenchymal tumors, these tumors exhibit high tumor cellularity and low infiltration of CD3+ and CD45+ stromal cells [111,112]. Proliferative subtype tumors are defined by an undifferentiated phenotype and express pro-proliferative signaling including increased expression of developmental transcription factors, proliferation markers, ECM-related genes, and WNT/β-catenin signaling, as well as increased expression of proteins involved in DNA replication [3,4,111,113]. In addition, it has been noted that these tumors express low levels of ovarian cancer marker genes (MUC1, MUC16, KLK6, KLK7, and KLK8) and high expression of the developmental transcription factors HMGA2 and SOX1. These tumors were also associated with an increased expression of FANC genes and homologous recombination [113,115]. 

Finally, differentiated subtype tumors have been shown to most closely resemble normal fallopian tissue at the gene expression level [111,115]. At the genetic level, these tumors are defined by increased expression of MUC1, MUC16, SLP1 (secretary fallopian tube marker), epithelial cell differentiation markers, and folliculogenesis-related genes which are indicative of increased tumor cell differentiation [3,113,115]. Proteomic analyses from the CPTAC project were able to further dissect signaling networks activated in these tumors to identify enrichment of protein expression programs associated with altered tumor cell metabolism and increased cell-to-cell communication [4] providing additional insight into subtype-specific mechanisms driving tumor development and progression.

Although these seminal studies were able to identify four largely concordant subtypes based on gene expression profiling, a number of recent studies have suggested that these subtypes are not consistent across platforms and populations [113,114,115,118]. These more recent studies have observed that tumors were able to be more robustly classified into fewer groups and/or that alternative strategies may provide additional insight into the underlying biology of this disease. Notably, studies from the CPTAC project were able to utilize proteome-wide data from 9600 proteins and 6769 phosphoproteins from 174 tumor samples to identify altered signaling networks in the transcriptome based subtypes further refining and validating the distinct signaling networks in these tumors, as well as identifying signaling pathways correlated with homologous recombination deficiency phenotype and patient survival [4]. However, in this proteogenomic analysis of ovarian tumors, Zhang et al. also identified five distinct protein-based subtypes and were able to show that three of the five subgroups were largely concordant with the TCGA mRNA-based subtypes. The remaining two subgroups represented tumors defined by unique underlying biology that would not be apparent by assessing mRNA data alone. Consistent with this premise, a number of recent studies have attempted to move beyond mRNA- or protein-based approaches to incorporate phosphoproteomic or glycoproteomic profiling to investigate the heterogeneity of HGSOC tumors [119,120]. These studies have provided additional depth to our understanding of HGSOC tumorigenesis by identifying subgroups defined by unique patterns of active kinases and altered cell signaling which contributing to tumor development, progression, and clinical outcome. Likewise, recent work by Karagoz et al. [116] assessed patterns of oncogenic signaling using a panel of 62 gene expression-based signatures across the four TCGA subtypes in three unique datasets [3,111,121]. As noted above, these studies identified unique oncogenic and tumorigenic signaling pathways associated with each mRNA-based subtype. However, in contrast to similar analyses in breast tumors which demonstrated clear differences in pathway patterns between the PAM50 subtypes, the distinctions amongst ovarian subtypes appeared to be more subtle and included increased intra-subtype heterogeneity [99,116]. 

Collectively, these data reinforce the premise that ambiguity in HGSOC subtype assignment could be a result of shared common biological underpinnings, the existence of intermediate subtypes, or biased by tumor cellularity and/or composition. As such, it is apparent that further refinement of the molecular subtypes, potentially through the incorporation of multiple genomic or proteomic platforms, may be necessary for these classification schemes to be clinically relevant. 

At the molecular level, HGSOC has been classified as a C-class malignancy (chromosomally unstable) that is defined by extensive structural variants [102]. Consistent with this classification, mutational profiling of HGSOC by the TCGA project using whole-exome sequencing has identified a limited number of significantly mutated genes that define this disease [3]. The most prominent among these is TP53 mutations which are evident in nearly all patients and are believed to arise early in the transformation process [3,44,122,123,124]. Beyond altered p53 signaling, transforming oncogenic mutations in PIK3CA, BRAF, KRAS and NRAS have been detected in HGSOC, albeit at low frequencies (<1%). Almost half of HGSOC tumors are characterized by homologous recombination (HR) deficiency through germline or somatic mutations in BRCA1/2 (20%), BRCA1 hypermethylation (11%), and/or dysregulation of other HR genes including PTEN, ATM or ATR, RAD51C, EMSY and Fanconi anemia genes [3]. While few significant mutations are apparent in HGSOC, DNA copy number alterations are more frequent in these tumors [3,102]. This includes amplification of MECOM, MYC, and CCNE1 which are among the most significant focal amplifications and found in more than 20% of HGSOC cases in addition to KRAS and MAPK1 which are found in more than 10% of cases. 

Interestingly, while specific genes are mutated at a low frequency in HGSOC, pathway analyses incorporating orthogonal whole-exome sequencing and copy number data demonstrated that HGSOC tumors are characterized by aberrant RB1/E2F (67%), PI3K/RAS (45%), and NOTCH (22%) signaling as well as dysregulation of the FOXM1 transcription factor network (87%) [3]. Further pathway analysis, based on phosphoproteomic profiles of HGSOC tumors demonstrated differential expression of RhoA-regulatory, PDFRB, and integrin-linked kinase pathways between poor and good prognostic HGSOC patients [4]. 

Finally, a number of recent studies have used integrative analyses to identify novel oncogenes and tumor suppressors that promote HGSOC and biomarkers to predict therapeutic response and risk. These studies relied on integrative analyses of DNA copy number, methylation, and gene expression data to identify potential oncogenes and tumor suppressor proteins in HGSOC and clear cell carcinoma [125,126,127]. Similarly, studies from Karagoz et al. assessed orthogonal genomic and proteomic data from human HGSOC tumors from the TCGA and CPTAC studies in the context of a prognosis gene expression signature. These analyses, along with data from a genome-wide RNAi screen in ovarian cancer cell lines, identified ADNP as a novel oncogene in HGSOC and in vitro studies showed that this protein regulates cell survival through altered cell cycle checkpoints [116]. While the therapeutic potential of these genes remains unclear, studies by Kurimchak et al. incorporated kinome profiling of human tumors and PDX models to identify MRCKA as a potentially drug-able oncogene activated in a subset of HGSOC tumors. Subsequent loss-of-function studies demonstrated that this gene could regulate HGSOC tumorigenesis and could be pharmacologically inhibited suggesting it may have potential as a novel therapeutic target [128]. Finally, studies from Coscia et al. identified CT45 as a biomarker for platinum-sensitivity in HGSOC using global proteomic profiling and demonstrated that mRNA or protein expression was associated significantly with chemosensitivity and disease-free survival [129].

## 5. Genetic and Genomic Relationship between Breast and Ovarian Tumors 

As discussed above, both familial and non-inherited breast and ovarian cancers have been shown to have similar genetic and genomic features (Figure 1). Beyond the previously discussed correlation between inherited mutations and the increased risk of breast or ovarian tumor development, analysis of human breast tumors demonstrated that HGSOC tumors also express a basal-like gene expression signature [2]. This relationship was further validated by multi-platform genomic analyses in which basal-like and HGSOC tumors were found to have a strong genomic association based on global mRNA profiling and to express a similar pattern of DNA copy number alterations [130]. While similar patterns of gene expression between these two diseases were noted by Hoadley and colleagues in a pan-cancer analysis of 12 tumor types and by the TCGA breast cancer paper, this association was not as clear when studied within the context of 33 tumor types potentially reflecting differences driven by tumor cell of origin, additional variability due to a more diverse tumor population, or other technical or biological factors [2,130,131]. Regardless, basal-like and high-grade serous ovarian tumors are classified as C-class malignancies and are characterized by predominant recurrent copy number alterations [102]. Specifically, these tumors share copy number gains of 1q, 3q, 8q, and 12p, and copy number losses of 4q, 5q, and 8p [2,100]. Among the commonly amplified genes are MYC (8p21.21), CCNE1 (19q13.2), MECOM (3q26.2), FGF3 (4p16.3), MCL1 (1q21.3) and ERBB3 (12q13.2) [43]. Additionally, basal-like and HGSOC tumors share RB1 loss in 20% and 10% of tumors, respectively [2,3]. 

Beyond copy number alterations, these tumor subtypes have been shown to express similar mutation profiles for a limited number of key oncogenes and tumor suppressor genes. Basal-like and high-grade serous ovarian tumors are enriched for BRCA1/2 inactivation and express TP53 mutations in 90–95% of tumors [2,3,5]. In addition, both tumor types exhibit an increased frequency of genome breakpoints as well as a loss of heterozygosity and allelic imbalance indicating genomic instability and homologous recombination deficiency [132,133,134]. More recent studies have indicated that these tumors demonstrate high homologous recombination deficiency (HRD) scores, accumulation of large-scale state transitions, increased loss of heterozygosity (LOH), and telomeric allelic imbalance scar signatures. Clinically, these alterations have been shown to be significantly correlated with pathologic complete response and minimal residual disease in TNBC patients treated with platinum-based therapies and with a better prognosis in HGSOC [36,135,136]. 

In addition to specific mutations and genomic alterations, basal-like breast and HGSOC tumors have been shown to express similar signaling networks including increased activation of PI3K signaling [2,3,102,137,138,139]. While PIK3CA mutations are relatively rare events in each tumor type, a number of unique alterations have emerged as contributing to aberrant signaling [2,3,5]. In HGSOC, DNA copy number gains in PIK3CA (18%), AKT1 or AKT2 (9% combined) and to a lesser extent, homozygous deletion of PTEN (7%) are the main drivers for this pathway [3,140,141]. In contrast, basal-like tumors are regulated by alterations in multiple genes (EGFR, IGFR1, AKT3) that occur at a low frequency (2–4%), as well as a loss of PTEN (35%) or INPP4B (30%), SOX4 amplification and overexpression, and MAGI3-AKT3 gene fusion [2,137,142,143]. Interestingly, these data indicate that while both tumor types are characterized by high PI3K signaling, the mutations activating signaling in each tumor type differed in prevalence and composition. 

Similarly, on a pathway activity level, basal-like and HGSOC tumors share increased FOXM1, HIF1-α, and MYC signaling [2]. Basal-like breast cancers have increased altered cell cycle checkpoint regulation, DNA damage repair, MYC, and immune response signaling [5], while proteins associated with recurrent copy number alterations in HGSOC converge on cell migration/invasion and immune regulation pathways [4]. Consistent with common alterations between basal-like and high-grade serous ovarian tumors, Marcotte and colleagues [144] used a genome-wide pooled shRNA screen in 29 breast and 15 ovarian cancer cell lines to identify genes uniformly essential for cell viability as well as genes required within each disease type. While cell line-specific genes were identified, these analyses also identified 66 ovarian and 155 breast cancer-specific genes as well as 297 genes that were essential for viability in the majority of cell lines irrespective of tissue type. While the latter set of genes did not necessarily take into account distinctions between molecular subtypes, these studies further reinforce the shared underlying biology of these diseases. 

## 6. Advances in Genomic Analyses of Breast and Ovarian Cancer

**Figure 2:**
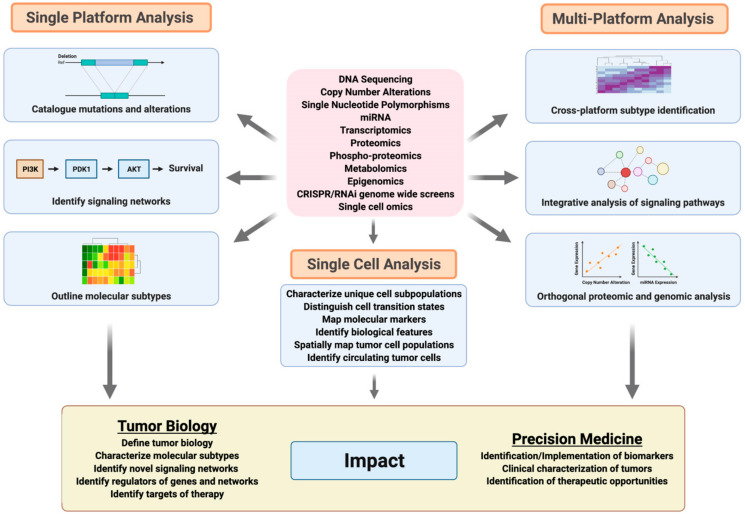
Use of single platform and integrative omics in cancer biology and medicine. The major contributions of single and multi-platform omics studies as well as single cell omics are summarized here. Single platform studies enable cataloging of mutation or alteration patterns, identifying signaling networks of interest and defining certain molecular subtypes. Multiplatform studies can further expand single platform-defined molecular subtypes and identify signaling pathways by identifying mutations in multiple genes representing multiple levels of pathway dysregulation. Single cell analyses allow for analyses of tumor cell subpopulations, identify cell transition states, map molecular markers and cell populations and identify circulating tumor cell populations. Orthogonal analysis of these data provides further context to genomic studies. These approaches contribute to a greater understanding of tumor biology as well as clinical advancements in treating cancer.

Single-platform genomic and proteomic analyses have allowed for the identification and cataloging of mutations; copy number alterations; and altered gene, miRNA, protein, or phosphoprotein expression profiles [2,3,7,12,23,145,146,147,148,149,150,151,152]. As we have outlined above, patterns of genomic and proteomic alterations can define tissue- and histological-specific differences in underlying biology and can be used to define molecularly distinct subtypes of cancer, including breast and ovarian cancer [2,3,11,23,97,118,119,120,136,153,154,155]. These genomic and proteomic patterns can identify oncogenic mechanisms that contribute to disease development, progression and in some instance can serve as therapeutic targets or markers of therapeutic response [10,62,63,99,102,113,126,128,129,135,137,142,156,157,158,159,160,161,162,163,164,165,166,167,168,169,170,171,172]. However, single platform analyses can be limited in their ability to visualize altered signaling networks and oncogenic processes. Given the complexity of mechanisms regulating these processes, multiplatform analyses, incorporating orthogonal genomic and proteomic data, enable the visualization of various types of alterations, in multiple key components within a given network to better define the state of signaling within specific tumors types and/or subtypes [102]. More importantly, integrative multiplatform analyses have led to the comprehensive identification of actionable alterations through reverse engineering of signaling pathways, while identifying upstream effectors and downstream targets using multiple omics platforms [156,173,174,175,176] (Figure 2). Ultimately, integrative analyses have resulted in the discovery of novel tumor-promoting mechanisms with higher confidence.

Breast and ovarian tumors are comprised of a complex collection of cell types including multiple populations of tumor cells, stroma, immune cells, fibroblasts, and other cells that encompass the tumor microenvironment [177,178]. As we have discussed, omics technologies that rely on analysis of the entire tumor (i.e., bulk analysis) have provided an enormous amount of insight into tumor biology; however, these approaches represent an averaged view of the tumor landscape and do not allow for fine resolution at the single cell level. Although a number of approaches including ESTIMATE, and others, have been developed to delineate specific signaling networks that arise from discrete cell populations or to estimate differences in cell composition within tumors using bulk sequencing or proteomic data, these methodologies are unable to fully address these challenges [179]. Advances in single-cell omics have had a significant impact on our understanding of tumor characteristics that are not apparent by bulk genomic, proteomic, or metabolomic approaches. These methods have allowed us to identify and characterize unique cell subpopulations, distinguish cell transition states, map molecular markers, identify novel and previously unrecognized biological features, and in combination with other technologies, are beginning to be used to spatially map tumor cell populations, identify circulating tumor cells and provide mechanistic insight into tumorigenic processes including metastasis and therapeutic response. Given spatial limitations, we point our readers to an excellent collection of review articles that discuss these advances in depth [180,181,182,183,184,185,186,187,188,189,190,191].

A number of recent studies have employed single-cell RNA-sequencing (scRNA-seq) analyses to examine tumor immune profiling [192,193,194,195,196,197]. These approaches have clear implications for both our understanding of the role of the immune system in the tumor microenvironment and for determining or predicting the efficacy of immunotherapy-based treatments. Of note, a recent study by Azizi et al. demonstrated the wide variability in immune cell type composition between breast cancer patient samples in addition to highlighting the phenotypic expansion of intratumoral immune cells using single-cell RNA and T cell receptor sequencing [198]. Further studies by Savas and colleagues demonstrated the association between tissue-resident memory T cell differentiation signature, developed using single-cell RNA-seq, and prognosis in early stage triple-negative breast [199]. Demonstrating the potential clinical implications and applicability of these approaches, investigators have used these technologies to identity mechanisms of therapeutic resistance [200]. Notably, recent work identified enrichment of immunosuppressive immature myeloid cells (IMC) in anti-Her2 and CDK4/6 inhibitor-resistant HER2-positive breast cancer, while combinatorial treatment with cabozantinib (IMC-targeting tyrosine kinase inhibitor) and immune checkpoint blockade overcame resistance [201]. Moreover, scRNA-seq has been used to develop gene-expression-signature of the myeloid-derived suppressor cells (MDSCs) in addition to identifying CD84 as a surface biomarker for MDSCs in breast cancer [202]. Similarly, Wan et al. reported reprogramming of inert natural killer and T cells to a highly active cytotoxic state following bispecific anti-PD-1orPD-L1 antibody treatment using single-cell RNA-seq analysis of HGSOC organoid co-cultures; this study identified a potential advantage of bispecific antibodies in immune checkpoint blockade therapy in HGSOC [203]. Further analyses have identified inter- and intra-tumor heterogeneity in cancer associated fibroblasts cell states in HGSOC and breast cancer [117,204]. Collectively, immune profiling coupled with imaging and single-cell RNA-seq underscored the importance of the spatial architecture of tumor niches in regulating immune infiltration and activation [205,206,207,208,209,210,211,212].

A number of recent studies have employed single-cell analyses to investigate inter and intra- tumoral heterogeneity [213,214,215,216,217,218]. Of note, recent work by Chung et al. linked tumor-intrinsic and immune cells diversity with TNBC intratumoral heterogeneity while studies by the Ellisen laboratory identified a connecting between intertumoral heterogeneity and clonality of inferred genomic copy number changes in these tumors. These latter studies suggested that cellular genotype drives gene expression programs, including signatures of treatment resistance and metastasis, in individual tumor cell populations [219,220]. Consistent with this premise, investigators have identified rare plastic pre-adapted cell subpopulations in luminal breast tumors which showed resistance to acute endocrine treatment [221]. Similarly, studies by Izar et al. and Geistlinger et al. used scRNA-seq based analyses to link the transcriptomic-based subtype classification of HGSOC to tumor cell type composition rather than intrinsic difference in gene expression patterns present in tumor epithelial cells further highlighting the importance of considering specific subpopulations of cells and the impact of signaling from the microenvironment on tumor characteristics [117,222]. More complex analyses integrating single-cell RNA-seq coupled with cell lineage tracing has been used to detail tumor cell subpopulations that contribute to various aspects of tumor evolution, including identifying pre-EMT (Epithelial to Mesenchymal Transition) cells that are essential for metastasis initiation [223].

Beyond assessing the transcriptome, single-cell DNA sequencing approaches have been developed and used to identify subpopulations of cells that express unique mutational and CNA patterns in therapeutically actionable genes in a given breast tumor [224]. These findings have clear clinical implications as different subpopulations will be likely be uniquely sensitive or resistant to specific therapeutic regimens and contribute to the evolution of the tumor and therapeutic sensitivity. Consistent with this premise, longitudinal sequencing analyses of tumors have demonstrated the emergence and/or re-emergence of clonal populations following treatment [225,226,227]. Complementary to these studies, single-cell mass cytometry using CyTOF identified rare tumor subtypes in HGSOC in addition to the dominant subsets and demonstrated that one of the identified rare subtypes was enriched for EMT signaling and associated with increased tumor metastasis [228]. Finally, merging single cell proteomics with other omics analysis has enable investigators to capture tumor-immune interactions in breast tumors [212]. Collectively, single-cell omics underscored intra- and inter-tumoral heterogeneity, identified subpopulation-specific vulnerabilities and emphasized the importance of addressing these vulnerabilities with combinatorial targeted therapeutic options [117,214,220,229,230,231,232,233].

Traditionally genome-wide RNAi and CRPSR/Cas9 screens have identified novel essential genes and pathways [144,234,235]. These studies evolved to include chemo-genetic screens which incorporate loss-of-function screens coupled with drugs or small molecule inhibitors in order to identify drug-gene interactions, cancer genetic vulnerabilities, and potential drug resistance mechanisms [21]. More recently, investigators have begun to incorporate these studies with multi-dimensional genomic analyses of human tumors as an added functional filter to identify clinically relevant cancer vulnerabilities and potential novel therapeutic targets. For instance, Marctotte and colleague integrated a pooled shRNA screen with genomic, transcriptomic and proteomic data from 77 breast cancer cell lines to identify breast cancer subtype-specific vulnerabilities. In this study, PSMB3, PSMA6 and ATP6V1B2 were identified as top ranked “basal-selective” essential genes [234]. Likewise, integrative correlative studies between pathway-specific gene expression signature scores, gene level DNA segment scores and RNAi shRNA abundance led to the identification of 21 amplified, essential and putative driver oncogenes in highly proliferative luminal breast cancers as well as the identification of SOX4 as a driver of PI3K signaling in basal-like breast tumors [99,137]. Similarly, in ovarian cancer, systemic loss-of-function shRNA screen identified 50 essential and amplified genes including CCNE1, PAX8, FRS2, PRKCE, and RPTOR. Of note, PAX8 was found to be amplified in 16% of primary ovarian cancers while shRNA mediated silencing of PAX8 lead to apoptosis in cell lines harboring either PAX8 amplification or overexpression [235]. Likewise, ubiquitin B (UBB) and ubiquitin C (UBC) were identified as a paralog deficiency dependency in ovarian cancers, implying the essentiality of UBC in cell lines with repressed UBB [166]; shRNA mediated silencing of UBC in UBB repressed ovarian cancer xenograft model lead to tumor regression and prolonged survival [160]. More recent studies have evolved to employ machine learning algorithms for predicting functional cancer vulnerabilities while integrating shRNA (DEMETER2) or CRISPR/Cas9 (DepMAp) screens coupled with genomic and proteomic profiling of cancer cell lines [236]. Collectively, genome-wide RNAi and CRPSR/Cas9 loss-of-function screens made a significant contribution in identifying cancer dependencies, and potential novel therapeutic targets [166,237,238,239].

Unfortunately, spatial limitations prevent an in-depth discussion of the many tools, algorithms, and computational approaches that have been developed for a single platform and integrative analyses. However, biochemical and genetic-based studies as well as large-scale proteogenomic analyses have demonstrated that despite enormous tumor heterogeneity, molecular alterations often converge on a limited number of signaling networks, reflecting pathway activity levels and their role in driving tumor progression [14,102,147,149,240,241]. As a result, a number of tools and approaches have been developed, including the use of gene expression-based signatures, mutational signatures, CNA (copy number alterations) signatures, and protein signatures to quantify pathway activity [7,96,97,242,243,244]. These approaches include several that incorporate data from multiple technical platforms and use statistical modeling driven by a priori knowledge of signaling pathways and/or protein–protein interaction networks to cluster samples based on similarity networks, detect enriched signaling networks across multi-omics platforms and/or infer pathway activity from the expression or mutation profiles of established pathway components [3,8,23,170,245,246].

As we have outlined, large-scale genomics and proteomics studies including those from the TCGA, METABRIC, and CPTAC projects, as well as many other studies, have enabled the cataloging molecular alterations and signaling pathways in breast and ovarian cancers. While these studies have implications for our understanding of the underlying molecular mechanisms of these diseases, they also highlight the need to personalize therapeutic approaches based on the biology of each patient’s disease [6,131,247]. Consistent with this premise, the use of genomic profiling, including DNA sequencing gene panels from Foundation Medicine and others, into clinical trials and practice has identified potential biomarkers, beyond standard immunohistochemistry (IHC)-based assays, to predict response and provided a means to personalize treatment. In addition to DNA sequencing-based assays, multiple molecular biomarkers are currently being used to monitor and track the progression of both ovarian and breast cancer [167,248,249]. For ovarian cancer patients, these biomarkers include single gene markers CEA (Carcinoembryonic Antigen), CA125 (Cancer Antigen 125), and HE4 (Human Epididymis protein 4), as well as multivariate index assays including OVA1, ROMA, and OVERA [250,251,252,253,254,255,256,257]. Similarly, several gene-expression based prognostic tests, including Oncotype DX [258], EndoPredict [259] and MammaPrint [260] as well as the aforementioned Prosigna assays, have been FDA approved to predict risk of recurrence in breast cancer and can be used to help guide clinical decisions. Oncotype DX Recurrence Score is based on the expression level of a panel of 21 genes, which is used to predict the likelihood of the 10-year tumor recurrence and guiding the adjuvant treatment options while weighing the added benefit of adjuvant chemotherapy versus treatment with hormonal therapy alone. Oncotype Recurrence Score stratified patient samples into low-, intermediate- and high-risk groups, predicting high likelihood of added benefit of adjuvant chemotherapy in the high-risk group patient cohort [258,261,262]. MammaPrint on the other hand is based on the gene expression profile of a panel of 70 genes. This test is used in clinics for assessing clinical outcome and predicting recurrence score in early stage breast cancer. Based on recurrence scores, patient samples are stratified into low or high genomic risk. Studies showed that there is an added benefit to adjuvant chemotherapy in the low genomic risk group when compared to patients who did not receive chemotherapy [260,263]. Finally, emerging data supports the role of analyses of circulating tumor DNA in routine clinical care [48,264,265,266,267,268]. The FDA recently approved the FoundationOne Liquid CDx test, which is a circulating cell-free DNA (cfDNA) based-assay as a companion diagnostic for treatment of BRCA mutant (germline or somatic) ovarian cancer patients with the PARP inhibitor rucaparib as well as alpelisib treatment of HR+/HER2-, PIK3CA mutated breast cancer patients [249]. 

## 7. Summary

Over the past twenty years, proteogenomic profiling of human tumors has drastically expanded our understanding of breast and ovarian cancer biology. The identification of molecular subtypes; novel oncogenes, tumor suppressor proteins and signaling networks; as well as clinically relevant biomarkers have begun to contribute to the development of novel and more effective treatment strategies. The next challenge will be to effectively translate these efforts into the development of new clinical diagnostic tools, biomarkers, and therapeutic strategies in order to personalize cancer treatment and improve the outcome and quality of life for patients. 

## Data Availability

Not applicable.
